# Do China's e-cigarette control policies work? A decade-long analysis of public discourse using an AI-integrated mixed-methods approach

**DOI:** 10.18332/tid/208810

**Published:** 2025-09-26

**Authors:** Zhangyan Li, Xinrui Wang, Xingye Yao, Yu Chen

**Affiliations:** 1 College of Media and International Culture, Zhejiang University, Hangzhou, China; 2 School of Journalism, Communication University of China, Beijing, China; 3 Department of Software Development, Fujian Minguang Software Co., Ltd. Sanming, China; 4 School of Art and Communication, Fujian Polytechnic Normal University, Fuzhou, China

**Keywords:** e-cigarette prevention, China’s policy, tobacco control, mix-research method

## Abstract

**INTRODUCTION:**

China, the world's largest tobacco market, has raised concerns due to e-cigarettes' health risks and rising youth usage. Despite a decade of regulatory policies, their effectiveness remains uncertain. This study examines trends in e-cigarette discourse on Weibo (2016–2025), analyzing discussion volume shifts and the impact of various topics on public engagement.

**METHODS:**

This study employs a hybrid computational approach integrating topic modeling, LLM-assisted annotation, and quantitative analysis to examine the evolution of e-cigarette discussions on Weibo (2015–2025) and topic dissemination effects (n=129769). LDA modeling identify 10 topics, followed by DeepSeek-V3-assisted classification. Linear regression in SPSS analyzed relationships between topic categories and social media engagement metrics (reposts/comments/likes) at 95% confidence intervals.

**RESULTS:**

Findings reveal 2020 as a key year of change: pro-vaping posts declined while anti-vaping content increased. Despite reduced volume, pro-vaping material maintained significant digital influence. Pre-policy, marketing content (p<0.01), health effects (p<0.01) and regulation (p<0.01) drove engagement. Post-policy, marketing lost engagement impact, while ‘user experience’ posts gained traction, significantly correlating with all interactions (all p<0.05). This indicates regulations were less effective against user-generated content, with pro-vaping messaging shifting towards peer-driven channels. Crucially, influencers consistently triggered strong engagement throughout the period (p<0.01) despite lower post volume, remaining key discourse drivers.

**CONCLUSIONS:**

Although China is strengthening its control over e-cigarettes, the results of our study indicate that this control remains limited. We advocate for more robust regulation of social media content, particularly concerning the management of celebrities and influencers, as well as the sharing of e-cigarette use experiences. However, the current regulatory framework enforced by the State Tobacco Monopoly Administration has proven inadequate for widespread and effective governance. We suggest that regulatory authority be shared with public health agencies in order to better integrate e-cigarette regulation with broader public health objectives.

## INTRODUCTION

Since the emergence of electronic nicotine delivery systems (ENDS), commonly known as e-cigarettes, in the mid-2000s, there has been growing public concern about their health effects and increasing usage rates, particularly among adolescents^1^. E-cigarettes are battery-powered devices that vaporize nicotine through heating rather than combustion and are often promoted as a safer alternative to traditional cigarettes^2^. However, studies have indicated that they may lead to nicotine addiction, respiratory problems, and increased risk of using traditional tobacco^3^. Globally, the use of e-cigarettes has surged, largely driven by aggressive marketing and widespread availability^4^. As the world’s largest tobacco market, China has seen a significant increase in the number of e-cigarette users, as evidenced by multiple studies^5-7^, prompting the government to introduce a series of regulatory policies on e-cigarettes over the past decade. Early regulatory attempts, such as the 2019 ban on online sales and advertising, were limited in effectiveness due to vague classification and weak enforcement^8^. From 2021 to 2022, the government incorporated e-cigarettes into the traditional tobacco regulatory framework, implementing licensing systems, technical standards, and tax regulations^9^.

However, despite the tightening regulations, previous studies have pointed out that the effectiveness of these policies remains questionable, especially under the structural contradiction in which the State Tobacco Monopoly Administration acts both as a regulator and a beneficiary of the tobacco industry, where this conflict of interest continues to undermine the actual effectiveness of tobacco control policies^10^. To evaluate the impact of these policies, especially on public perceptions of e-cigarettes, it is necessary to observe changes in public discourse. In China, Weibo is one of the largest social media platforms and serves as a major space for public discussion. However, existing studies rarely focus on the temporal evolution of public attitudes toward e-cigarettes on this platform, mostly centering on awareness or information exposure effects^11,12^, without tracking public opinion trends or whether they align with national policy narratives – particularly after the implementation of stricter regulations in 2021.

This study conducts a ten-year longitudinal analysis to fill the above research gap. Based on agenda-setting theory, we hypothesize that after policy implementation, pro-e-cigarette content should decrease while anti-e-cigarette content should increase. Although previous research has pointed out a shift toward negative public sentiment, most studies stopped at 2019 and mainly focused on changes in word frequency, failing to further examine discourse structures and narrative transformations^13^.

Furthermore, despite stricter regulations, e-cigarettes remain popular, suggesting that surface-level changes may not indicate substantive policy impact. Social media is increasingly weakening traditional media’s agenda-setting power, constructing a more pluralistic public sphere in which users, opinion leaders, and interest groups co-construct narratives. In such a media environment, state-led policy frameworks may struggle to maintain discursive dominance, especially on participatory platforms such as Weibo^14^.

This study analyzes Weibo data from 2015 to 2025 to examine the evolving discourse and shifting influence of topics related to e-cigarettes. By tracing these transformations, the research aims to reveal the effects of China’s tobacco control policies on the dynamics of public opinion dissemination.

## METHODS

### Research design

This study adopts a hybrid computational research design that integrates topic modeling, large language model (LLM)-assisted annotation, and conventional quantitative analysis. Our objective is to explore the evolution of public discourse on e-cigarettes on the Chinese social media platform Weibo from 2015 to 2024, as well as to assess the communicative effectiveness of different topics. The study is observational and non-interventional in nature, and does not aim to establish causal relationships.

### Data collection

We initially retrieved 129769 Weibo posts containing the keyword ‘e-cigarette’ between 28 February 2015 and 28 February 2025. After removing duplicates and filtering out entries lacking essential metadata – such as textual content, user ID, geolocation, identity attributes, or engagement indicators – we retained a refined dataset of 91750 valid posts, spanning from 1 January 2016 to 31 December 2024. This curated dataset formed the basis for both topic modeling and LLM-assisted content annotation.

### Variable definitions and annotation strategy

To address the thematic and framing structures of e-cigarette discourse, we employed a mixed approach combining Latent Dirichlet Allocation (LDA) and LLM-based classification. LDA, an unsupervised probabilistic model, assumes that each document is a mixture of latent topics, and that each topic is represented by a distribution over words. Although we tested alternative models such as Biterm Topic Modeling (BTM) and neural topic models, LDA was ultimately selected for its interpretability and robustness.

Based on prior research experience, we hypothesized the optimal number of topics to be between 5 and 50. LDA models were trained at 5-topic intervals, and model performance was evaluated using perplexity and coherence scores. Perplexity indicates the model’s predictive performance, while coherence reflects the semantic interpretability of topics. After comparing results across models, we selected a 10-topic solution (perplexity=0.0002; coherence=0.5272) as the basis for further classification.

Rather than relying solely on LDA’s probabilistic outputs, which may neglect word order and context, we enhanced classification accuracy by incorporating LLM-based zero-shot annotation. Prompt engineering was used to guide the model’s understanding of category definitions and contextual cues. Prior studies have shown that LLMs can achieve performance comparable to supervised models in zero-shot classification tasks, particularly when the number of categories is limited^15^.

Among various LLMs, we selected DeepSeek-V3 as the annotation backbone, due to its strong performance in Chinese-language classification tasks. DeepSeek-V3, with 671B parameters, has demonstrated excellent generalization and reasoning capabilities. According to Liu et al.^16^, DeepSeek-V3 outperformed GPT-4 in binary sentiment classification (99% vs 87.9%) and showed superior accuracy (81.3%) and recall in topic classification tasks, indicating its capacity to retrieve more relevant instances per category^17^.

In the meantime, to assess the reliability of automated annotations in this task, we conducted a validation test comparing the LLM’s outputs with those of two independent human coders on a random sample of 100 posts. The agreement rate reached 97% and 96%, supporting the reliability of using DeepSeek-V3 to annotate the remaining dataset. Each record was ultimately labeled with the following variables:

- Independent variable: Topic category (based on the 10-topic LDA classification)
- Dependent variables: Social media engagement metrics (repost count, comment count, like count)

### Statistical analysis

This study adopts linear regression analysis to examine the linear relationships and predictive power between the independent variables and the dependent variable. The analysis was conducted using IBM SPSS Statistics software, following the steps below.

*Data preparation and assumption testing*

The research data were imported into SPSS, and missing values and outliers were identified and addressed (e.g. using descriptive statistics or boxplots). Before conducting regression analysis, the distribution of the dependent variable was examined to assess whether it approximates a normal distribution. In SPSS, this was done by generating a histogram of the dependent variable and overlaying a normal curve, with results indicating a normal distribution.

*Model construction*

A linear regression model was established with the confidence interval set at 95% CI generated for each regression coefficient. A histogram of standardized residuals was plotted to test the assumption of normality of residuals.

*Multiple comparison correction*

When multiple independent variables are included in the model, simultaneously testing multiple regression coefficients increases the risk of Type I error (false positives). To control the overall error rate, this study applied a multiple comparison correction method by dividing the significance level α by the number of independent variables (k) tested in the model.

*Software and tools*

The tools included: Python version: 3.12.5; openai 1.68.2 (Volcano Engine API client); pandas 2.2.3 (data manipulation); openpyxl 3.1.5 (Excel handling) numpy 1.26.4 (numerical operations); jieba 0.42.1 (Chinese word segmentation); matplotlib 3.9.0 (data visualization); gensim 4.3.3 (topic modeling and text mining); and IBM SPSS Statistics Version 27.4 (for regression analysis and hypothesis testing)

## RESULTS

### Descriptive findings: Changes of topic on Weibo

LDA topic modeling identified five major themes comprising ten subtopics in Weibo’s e-cigarette discourse. The most frequently discussed theme was e-cigarette information (n=32379), which included topics such as tobacco control policies and legislation (n=12760), smoking and health effects (n=11419), and tobacco market and sales regulation (n=8200). These discussions reflect strong public concern about health risks and government regulation.

The second most prominent theme was e-cigarette promotion (n=29058), primarily consisting of user experiences (n=18227), followed by content on e-cigarette sales (n=8770) and flavors (n=2061). This category indicates a highly consumer-oriented discourse shaped by personal sharing and peer recommendations.

Drugs and anti-narcotics ranked third (n=25148), with a relatively even distribution between anti-drug enforcement and public campaigns (n=13072) and discussions on drug types and harms (n=12076), often linking e-cigarettes to broader drug control narratives.

**Figure 1:**
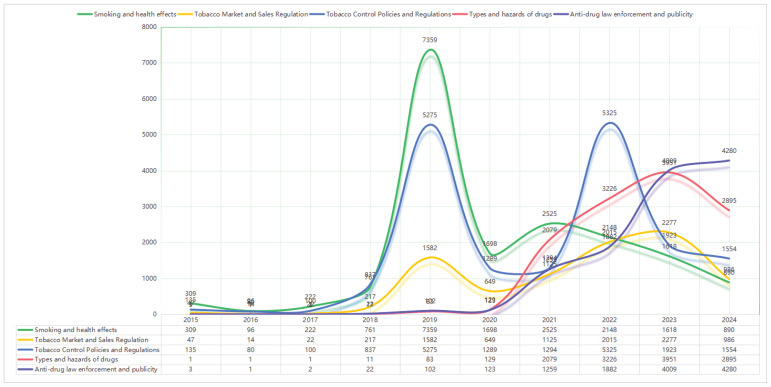
Anti-e-cigarette topic trends from 2015–2024

**Figure 2:**
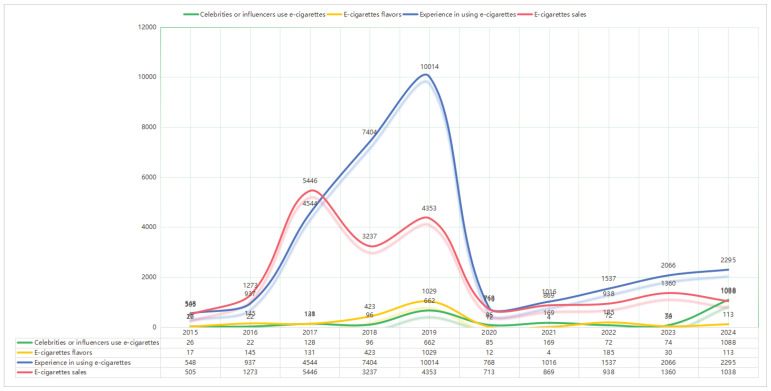
Pro-e-cigarette topic trends from 2015–2024

Entertainment and celebrity use was less prominent (n=3305), focusing on public figures and influencers using e-cigarettes. International news and policy was the least discussed theme (n=1860), covering global health events such as vaping-related illnesses and foreign regulatory responses (Table 1, Figures 1 and 2).

**Table 1:** Annual distribution of number of posts across topics (2016–2025)

*Topics*	*2016*	*2017*	*2018*	*2019*	*2020*	*2021*	*2022*	*2023*	*2024*	*Total*
Use of e-cigarettes by celebrities or influencers	22	128	96	662	85	169	72	74	1088	2422
Health effects of smoking	96	222	761	7359	1698	2,525	2,148	1618	890	17626
Tobacco marketing and sales regulation	14	22	217	1582	649	1125	2015	2277	986	8934
Tobacco control policies and regulations	80	100	837	5275	1289	1294	5325	1923	1554	17812
Flavors of e-cigarettes	145	131	423	1029	12	4	185	30	113	2089
Experience of using e-cigarettes	937	4544	7404	10014	768	1016	1537	2066	2295	31129
E-cigarette marketing and sales	1273	5446	3237	4353	713	869	938	1360	1038	19732
Types and harms of illicit drugs	1	1	11	83	129	2079	3226	3951	2895	12377
Anti-drug law enforcement and campaigns	1	2	22	102	123	1259	1882	4009	4280	11683
International relations and health issues (e-cigarette illness)	6	0	43	329	391	1031	291	60	55	2210

A comprehensive examination of the time trends from 2016 to 2024 shows that different subtopics related to e-cigarettes exhibited distinct developmental trajectories. These changes broadly align with our expectations: as policies evolved, the volume of various topics underwent significant shifts. The period around 2020 marked a critical turning point – before this, pro-e-cigarette discourse, such as vaping experiences and e-cigarette sales, showed a clear mainstream trend. However, after 2020, these discourses noticeably declined, while previously marginal anti-e-cigarette discourse began to rise around 2020 and had become dominant by 2024.

In more detail, topics related to national regulation and public governance –particularly drug control enforcement and tobacco control policies – showed a significant upward trend over the observation period. Mentions of anti-drug campaigns surged dramatically, reaching a peak of 4280 in 2024. This is an interesting finding, as drugs are a serious issue in China, and the metaphorical association of e-cigarettes with drugs indicates a growing public concern about the link between vaping products and illicit substances. Similarly, discussions about tobacco control legislation also steadily increased, peaking in 2022 – the same year that e-cigarettes were officially brought under the national tobacco monopoly framework. This suggests that our basic hypothesis is correct: as policies advance, related discussions are increasingly prevalent. These can be viewed as anti-e-cigarette discourses growing in tandem with the implementation of stricter policies.

Beyond formal governance, related topics concerning health and market regulation also experienced moderate growth. Discussions about the health impacts of e-cigarettes peaked in 2019, likely triggered by global conversations around vaping-related illnesses. However, this concern did not intensify in the following years. Attention to market regulation was more intermittent but saw a rise in 2022, coinciding with reforms in production and licensing. Although the growth of these two topics was neither dramatic nor sustained, they can still be seen as part of the strengthening anti-e-cigarette discourse over time.

In stark contrast to these strategic frameworks, user-generated and consumer-oriented topics showed stagnation or decline. Mentions of user experience peaked at 7404 in early 2018 and then gradually declined, indicating that public expressions of personal vaping use were fading from the public sphere. Discussions about e-cigarette sales followed a similar trajectory, peaking in 2019 before dropping sharply, likely due to restrictions on advertising and e-commerce. This is a positive sign, suggesting that the Chinese government’s policies have had a visible effect – suppressing pro-e-cigarette information on social media, particularly advertising content and personal experience sharing, which is significant for tobacco control efforts.

Additional pro-e-cigarette information includes posts about flavors and celebrity use, which showed more fragmented trends. Mentions of flavors grew slightly before 2019 but declined afterward – possibly due to policy emphasis on banning youth-oriented flavoring. Meanwhile, celebrity involvement remained relatively low for most of the past decade but surged in 2024, suggesting that celebrity vaping behavior may evolve into high-profile public events that attract considerable attention in a short time.

Finally, although international topics – such as global health incidents and foreign policy responses – briefly became focal points in 2021, their overall influence on long-term discourse remained limited. We believe this topic was primarily related to COVID-19 in 2021. China persistently promoted the notion of the ‘vaping illness’, implying that similar illnesses had circulated in the US before the virus was discovered in Wuhan. This issue was not directly relevant to e-cigarette regulation.

In summary, our data show that with the intervention of national policies, the discourse around e-cigarettes on Chinese social media indeed underwent an effective transformation. Pro-e-cigarette voices were suppressed, while anti-e-cigarette voices increasingly gained prominence. The turning point occurred around 2021, coinciding with the Chinese government’s implementation of more stringent regulatory measures.

### Correlation analysis

The descriptive statistics are shown in Table 2.

**Table 2:** Descriptive statistics (N=126014)

	*Min*	*Max*	*Mean*	*SD*	*Variance*	*Skewness*	*SE*	*Kurtosis*	*SE*
Reposts	0	549864	13.81	1598.738	2555961.82	325.771	0.007	111206.288	0.014
Comments	0	30424	9.28	168.188	28287.091	92.291	0.007	12082.046	0.014
Attitudes	0	700351	58.49	2381.607	5672050.055	216.209	0.007	60212.37	0.014
Use of e-cigarettes by celebrities or influencers	0	1	0.02	0.137	0.019	7.004	0.007	47.05	0.014
Health effects of smoking	0	1	0.14	0.347	0.12	2.077	0.007	2.312	0.014
Tobacco marketing and sales regulation	0	1	0.07	0.257	0.066	3.344	0.007	9.182	0.014
Tobacco control policies and regulations	0	1	0.14	0.348	0.121	2.059	0.007	2.239	0.014
Flavors of e-cigarettes	0	1	0.02	0.128	0.016	7.572	0.007	55.342	0.014
Experience of using e-cigarettes	0	1	0.25	0.431	0.186	1.173	0.007	-0.624	0.014
E-cigarette marketing and sales	0	1	0.16	0.363	0.132	1.89	0.007	1.572	0.014
Types and harms of illicit drugs	0	1	0.1	0.298	0.089	2.7	0.007	5.29	0.014
Anti-drug law enforcement and campaigns	0	1	0.09	0.29	0.084	2.809	0.007	5.889	0.014
International relations and health issues (e-cigarette illness)	0	1	0.02	0.131	0.017	7.351	0.007	52.04	0.014

SE: standard error.

*Extreme engagement polarization*

User interaction metrics exhibited severe positive skewness (skewness >90) and leptokurtic distributions (kurtosis >11000). Reposts (mean=13.81, SD=1598.738), comments (mean=9.28, SD=168.188), and likes (mean=58.49, SD=2381.607) demonstrated asymmetric viral diffusion, where minimal engagement characterized most content (minimum = 0) while outliers achieved disproportionate visibility (maximum reposts = 549864).

*Imbalanced thematic coverage*

Binary-coded topics showed significant disparities: Core discussions focused on e-cigarette experiences (mean=0.25), tobacco control policies (mean=0.14), and sales regulation (mean=0.16).

Persuasive elements like celebrity endorsements (mean=0.02, SD=0.137), flavor appeals (mean=0.02, SD=0.128), and international health issues (mean=0.02) remained critically underutilized drug-related topics occupied intermediate prevalence (hazards mean=0.10; anti-drug campaigns mean=0.09).

In the pre-2020 data, health effects of smoking showed a significant correlation with repost volume (p<0.05). Use of e-cigarettes by celebrities or influencers (p<0.01), tobacco control policies and regulations (p<0.01), and e-cigarette marketing and sales (p<0.01) were significantly correlated with comment engagement. Use of e-cigarettes by celebrities or influencers (p<0.05) and health effects of smoking (p<0.01) demonstrated significant correlations with like counts (Table 3).

**Table 3:** Correlation analysis of pre-2020 data

*Model*	*B*	*SE*	*β*	*t*	*Sig.*	*95% CI for B*		*Collinearity* *tolerance*	*VIF*
						*Lower*	*Upper*		
**Repost**									
Health effects of smoking	61.98	28.498	0.01	2.175	0.03	6.123	117.837	0.856	1.168
**Comment**									
Use of e-cigarettes by celebrities or influencers	32.511	5.446	0.025	5.97	0	21.837	43.184	0.977	1.023
Tobacco marketing and sales regulation	7.838	2.298	0.015	3.411	0.001	3.334	12.342	0.882	1.134
E-cigarette marketing and sales	-5.623	1.713	-0.015	-3.282	0.001	-8.98	-2.265	0.82	1.219
**Attitude**									
Use of e-cigarettes by celebrities or influencers	73.498	25.764	0.012	2.853	0.004	23	123.995	0.977	1.023
Health effects of smoking	38.227	9.674	0.018	3.951	0	19.266	57.189	0.856	1.168

B: unstandardized coefficient. β: standardized coefficient.

In the post-2020 data, experience of using e-cigarettes (p<0.05) and international relations and health issues (e-cigarette illness) (p<0.01) showed significant correlations with repost volume. Use of e-cigarettes by celebrities or influencers (p<0.01), experience of using e-cigarettes (p<0.01), and international relations and health issues (e-cigarette illness) (p<0.01) demonstrated significant correlations with comment engagement. Use of e-cigarettes by celebrities or influencers (p<0.01), Experience of using e-cigarettes (p<0.05), and international relations and health issues (e-cigarette illness) (p<0.01) exhibited significant correlations with like counts (Table 4).

**Table 4:** Correlation analysis of post-2020 data

*Model*	*B*	*SE*	*β*	*t*	*Sig.*	*95% CI for B*		*Collinearity* *tolerance*	*VIF*
						*Lower*	*Upper*		
**Repost**									
Experience of using e-cigarettes	18.637	7.698	0.011	2.421	0.015	3.549	33.725	0.694	1.44
International relations and health issues (e-cigarette illness)	102.453	13.266	0.031	7.723	0	76.451	128.455	0.895	1.118
**Comment**									
Use of e-cigarettes by celebrities or influencers	40.741	4.723	0.035	8.626	0	31.483	49.998	0.912	1.096
Experience of using e-cigarettes	10.466	2.503	0.019	4.181	0	5.56	15.372	0.694	1.44
International relations and health issues (e-cigarette illness)	45.796	4.314	0.043	10.616	0	37.341	54.251	0.895	1.118
**Attitude**									
Use of e-cigarettes by celebrities or influencers	671.993	87.047	0.031	7.72	0	501.381	842.604	0.912	1.096
Experience of using e-cigarettes	102.478	46.131	0.01	2.221	0.026	12.062	192.893	0.694	1.44
International relations and health issues (e-cigarette illness)	890.046	79.499	0.046	11.196	0	734.228	1045.865	0.895	1.118

B: unstandardized coefficient. β: standardized coefficient.

## DISCUSSION

This study examined changes in the volume and impact of e-cigarette discourse on Chinese social media over the past eight years. Our findings indicate that around 2020, coinciding with the implementation of regulatory policies, a discursive shift occurred on Weibo: pro-e-cigarette content declined while anti-e-cigarette narratives increased. However, despite the reduced volume of pro-e-cigarette posts, such content continued to wield significant influence in the digital space. The year 2020 emerged as a cognitive watershed in the online dissemination of e-cigarette-related content. Prior to the introduction of strict laws, marketing content strongly correlated with comment activity (all p<0.01), while topics like health effects (p<0.01) and tobacco regulation (p<0.01) also attracted substantial engagement through likes and comments. After policy enforcement, marketing-related content no longer triggered widespread interaction. However, user-experience-driven posts began to attract increasing engagement, showing significant associations with likes (p<0.05), reposts (p<0.05), and comments (p<0.01). This indicates that regulatory efforts were less effective in curbing the spread of user-generated content, with pro-e-cigarette messaging shifting from overt marketing to more personalized, peer-driven channels. Lastly, social influencers remained a key driver of e-cigarette discourse. Although their post volume was relatively low, they consistently triggered strong engagement across the entire study period (p<0.01).

This study provides important insights into the evolution of discussions related to e-cigarettes in China and highlights several deficiencies within the current regulatory framework. The value of our research lies first in offering a deeper level of analysis while confirming the findings of previous studies. The topic clustering in our study verifies earlier conclusions: as policies have been implemented, discussions about e-cigarettes on Chinese social media have undergone a welcome transformation. Pro-e-cigarette voices such as advertisements are decreasing, while opposing voices are increasing^13^. This is consistent with multiple previous studies. Our research has shown that 2019–2020 was a critical turning point for e-cigarette regulation in China. On 30 October 2019, the State Tobacco Monopoly Administration and the State Administration for Market Regulation jointly issued the first public notice on protecting minors from e-cigarettes, explicitly prohibiting tobacco advertisements targeting minors and strengthening content review in film and television^18^. On 1 November 2020, regulation was further intensified with a new notice requiring a complete shutdown of all online e-cigarette sales channels, including e-commerce storefronts and online advertising^19^. The accompanying policy interpretation specifically emphasized that the e-cigarette market at the time was in serious disorder, with safety hazards such as substandard batteries and e-liquid leakage, and with companies illegally adding various additives to attract consumers, especially minors – issues urgently requiring regulation. We believe this directly contributed to the observed shift in public discourse on Weibo.

However, our quantitative findings tell another story, suggesting that the situation may not be as optimistic as it seems. Pro-e-cigarette voices still maintain strong communicative power. This may indicate that the effects of Chinese policies are more superficial – achieved through mechanisms of discourse control – rather than representing real changes in public perception or a true suppression of pro-e-cigarette narratives. One piece of supporting evidence is the mutual confirmation between our study and that of previous research^20^, who found that the main characteristics of e-cigarette marketing on Weibo include emphasizing attractive product features, using popular figures, implicit promotions, downplaying health concerns, and engaging users in various ways. Together, our findings suggest that while the quantity of pro-e-cigarette content is declining, its influence remains.

Our study also confirms previous findings regarding the important role played by celebrities and internet influencers in shaping the use and promotion of e-cigarettes^21^. Our results show that interactions between celebrities/influencers and e-cigarettes generate significant communicative effects. This trend aligns with findings in international contexts, where influencer marketing has been shown to contribute to youth uptake of e-cigarettes. However, Chinese law does not prohibit public figures from using e-cigarettes^22^. Therefore, how to reduce the visibility and influence of such behaviors remains an important issue.

These two findings point to a shared concern: the need to strengthen regulatory interventions within China’s social media sphere. Yet, the structural configuration of tobacco governance in China makes this challenge more complex^23^. In 2021, the Ministry of Industry and Information Technology (MIIT) released a draft amendment to the Regulations on the Implementation of the Tobacco Monopoly Law of the People’s Republic of China, proposing to bring new tobacco products like e-cigarettes under the same regulatory framework as traditional cigarettes. However, regulatory authority over e-cigarettes still resides with the State Tobacco Monopoly Administration. The STMA currently holds a dual role as both an industry stakeholder and a regulatory body, creating a fundamental conflict of interest. Entrusting e-cigarette regulation to an institution historically responsible for managing and protecting the economic interests of the tobacco industry raises serious concerns about the legitimacy and effectiveness of such regulation. Moreover, the STMA lacks the capacity to regulate media – especially social media platforms that are privately owned – making it unlikely to effectively control the spread of content on these platforms^11^.

In this regard, the Beijing Tobacco Control Association stressed the urgent need to strengthen regulation of the e-cigarette market. It recommended transferring regulatory responsibility away from the tobacco monopoly system. Instead, it advocated for assigning this authority to public health agencies such as the National Health Commission, the National Medical Products Administration, or the State Administration for Market Regulation, to better align e-cigarette regulation with public health goals^24^. However, several years have passed since this proposal was made, and such advocacy has received little response. Our study further underscores that the current regulatory framework has failed to effectively curb the proliferation of e-cigarettes. Echoing the call from the Beijing Tobacco Control Association, we believe it is both necessary and urgent to transfer regulatory authority to health agencies. Such a shift would allow e-cigarettes to be governed primarily as a public health issue, rather than an economic or industrial one. Only within a health-centered regulatory framework can e-cigarette content be treated with the seriousness of other forms of health misinformation and be systematically regulated. This approach would not only enable better control over traditional forms of advertising, but also effectively address the informal and fragmented channels through which e-cigarette narratives continue to circulate.

We further call for the establishment of a coordinated regulatory alliance between public health authorities and digital platforms. Existing research shows that platforms often serve as dual intermediaries between user-generated content and national policies. Therefore, securing the cooperation of social media platforms is crucial for limiting the spread of pro-e-cigarette content. By implementing algorithmic filtering, content labeling, and co-regulatory mechanisms, platforms can play a proactive role in regulating e-cigarette discourse and supporting public health goals. This approach resonates with the Chinese government’s current emphasis on the concept of ‘collaborative governance’^25^, which aims to achieve more effective governance through interdepartmental cooperation. We endorse this concept and look forward to the development of a more comprehensive information governance framework within the realm of tobacco control – one that can weaken the communicative impact of e-cigarette marketing and experiential narratives.

### Strengths and limitations

This study has several key strengths. By integrating topic modeling, large language model (LLM) technologies, and quantitative analysis, we employed a mixed-methods approach to map the temporal evolution and communicative effectiveness of e-cigarette discourse. This multidimensional framework not only captures topic shifts over the past decade but also introduces a novel analytical lens – communication impact analysis – which has been largely absent in previous research. As such, our methodological paradigm is adaptable and can be applied to e-cigarette discourse in other national and regional contexts, offering comparative insights across different sociopolitical environments.

Nonetheless, several limitations must be acknowledged. First, the study does not adopt a causal design, limiting its ability to determine whether Chinese government policies have truly changed public perceptions of e-cigarettes beyond observable shifts in discourse. Future research incorporating causal frameworks would be beneficial. Additionally, our findings are based on the Chinese internet and media system, and therefore may have limited generalizability to other national contexts.

In terms of computational scope, a key limitation lies in platform selection: this study focuses exclusively on textual data from Weibo. In recent years, however, a substantial portion of e-cigarette-related content has migrated to image- and video-based platforms such as Xiaohongshu and Douyin (the Chinese version of TikTok). As a result, our longitudinal findings may underestimate the breadth and intensity of public discourse, particularly among younger users who primarily engage through these multimodal platforms. To address this gap, future studies should adopt cross-platform, multimodal approaches that incorporate visual and audiovisual data.

Lastly, residual confounding and selection bias may weaken the validity of causal inference. For complex sampling structures (e.g. multistage stratified sampling), SPSS’s weighted regression capabilities do not automatically integrate design weights, potentially disconnecting statistical inference from the target population. While SPSS’s GLM supports fixed effects, its mixed model (random effects) procedures are relatively complex and limited in interpreting cross-level interactions, making it difficult to control for macro-level confounders.

## CONCLUSIONS

Although China is strengthening its control over e-cigarettes, the results of our study indicate that this control remains limited. We advocate for more robust regulation of social media content, particularly concerning the management of celebrities and influencers, as well as the sharing of e-cigarette use experiences. However, the current regulatory framework enforced by the State Tobacco Monopoly Administration has proven inadequate for widespread and effective governance. We suggest that regulatory authority be shared with public health agencies in order to better integrate e-cigarette regulation with broader public health objectives.

## Data Availability

The data supporting this research are available from: https://github.com/w8692736/Electronic-Cigarette-Public-Opinion-Analysis-System
